# Retinal giant cyst treated by the scleral buckling procedure: A case report

**DOI:** 10.1097/MD.0000000000037620

**Published:** 2024-03-29

**Authors:** Lifei Wang, Yifan Wang, Xiaolu Cao, Peipei Jia, Lifei Yuan, Yanhui Wang, Jianan Liu, Jing An

**Affiliations:** a Department of Ophthalmology, Hebei Medical University, Shijiazhuag, Hebei, China; b Hebei Eye Hospital, XingTai, Hebei, China.

**Keywords:** retinal cyst, retinoschisis, scleral buckling

## Abstract

**Introduction:**

Retinal cysts are rare lesions of the fundus that are essentially fluid-filled cavities located or originating in the retina, with a diameter larger than the normal retinal thickness. To date, there have been few case reports of giant retinal cyst hemorrhage with retinoschisis.

**Case presentation:**

A 32-year-old woman with no other medical history complained of decreased vision for 3 days after a severe cough. The best-corrected visual acuity in the right eye was 0.5. A comprehensive ophthalmological examination including slit-lamp fundoscopy, ultrasound scan of the eye, optical coherence tomography scan, and orbital magnetic resonance imaging was performed. Ophthalmological examination revealed grade III anterior chamber blood cells and grade III vitreous hemorrhage in the right eye and a large herpetic cyst on the nasal side of the retina. The cyst projected into the vitreous, with a large amount of hemorrhage vaguely visible within it. The cyst was clearly visible, and a superficial retinal limiting detachment was observed around it. Ultrasound showed a retinal cyst with retinal detachment in the right eye. Laboratory test results were unremarkable. After 3 months of conservative treatment, the patient’s intracystic hemorrhage was significantly absorbed, but the size of the cyst cavity did not show any significant change. Scleral buckling with external compression combined with external drainage of the intracystic fluid was performed, the patient’s visual acuity was gradually restored to a normal 1.0 after the operation, and the retina appeared flattened. The patient was finally diagnosed with a giant retinal cyst with retinoschisis in the right eye. The presumed cause was heavy coughing leading to rupture and hemorrhage of the retinal cyst, similar to the mechanism of rupture of an arterial dissection. To the best of our knowledge, this case of retinal cyst rupture and hemorrhage caused by heavy coughing with good recovery after external surgical treatment has never been reported before.

**Conclusions:**

Giant cystic retinal hemorrhage with retinoschisis is very rare. Orbital magnetic resonance imaging and ocular B-scan ultrasound are essential for its diagnosis, and the selection of an appropriate surgical procedure is necessary to maximize the benefit for affected patients.

## 1. Introduction

Retinal cysts are usually located in the outer plexiform layer of the retina, separating its inner and outer nuclear layers.^[1]^ Most researchers believe that retinal cysts are secondary to long-term retinal detachment.^[2]^ This case report describes a rare large retinal cyst with hemorrhage caused by severe coughing, with limited retinal detachment with retinoschisis around the cyst, which was treated by scleral buckling with a good recovery.

## 2. Case presentation

**Figure 1.**
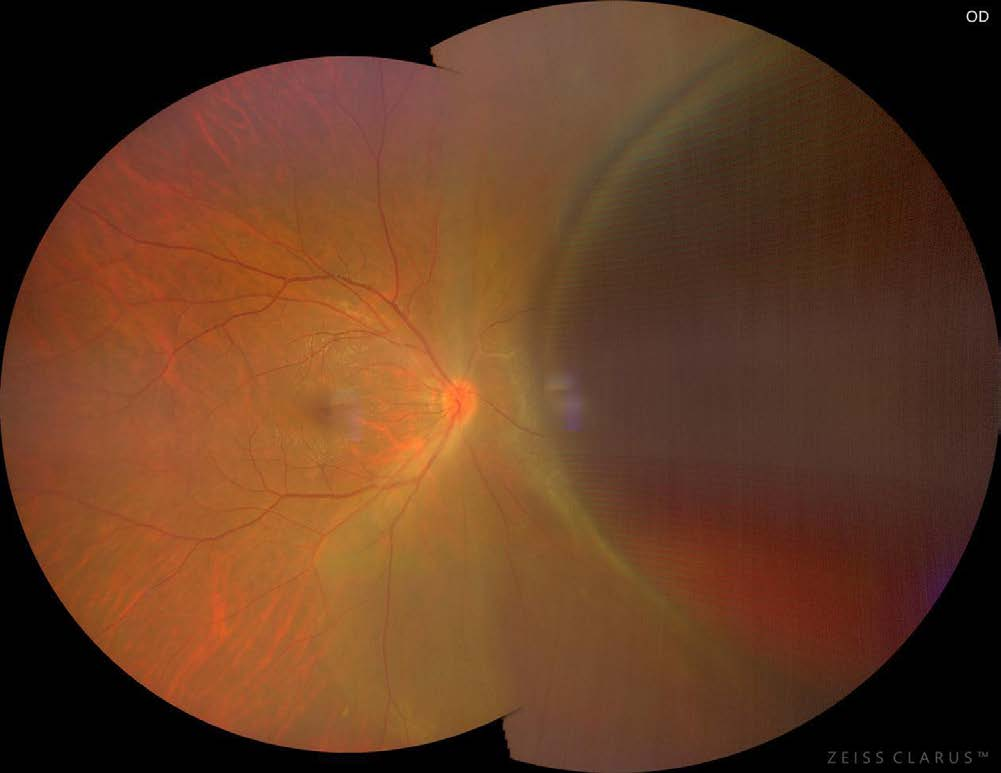
A large cyst was visible on the nasal side of the retina, protruding into the vitreous cavity, with well-defined borders, peripheral retinal limitation of superficial detachment, and a large amount of hemorrhage vaguely visible within the cyst.

**Figure 2.**
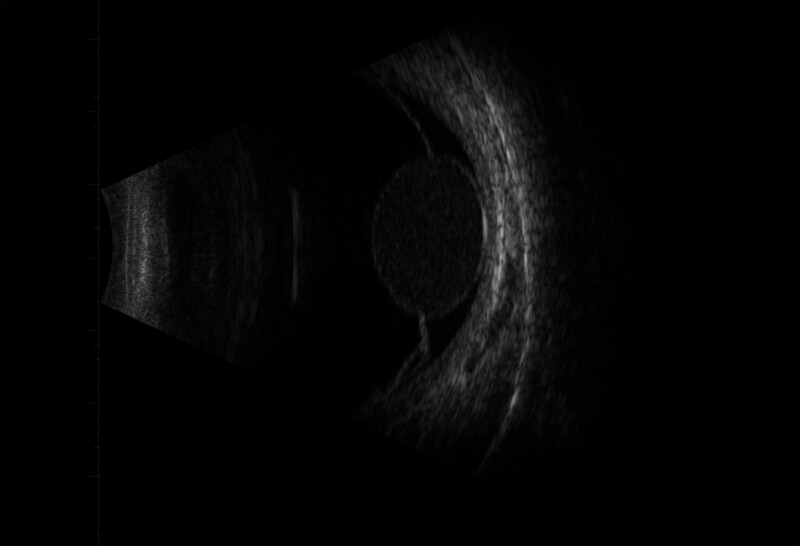
A B-scan image of the left eye demonstrated. (A) dense vitreous hemorrhage and (B) an area of superior nasal bullous retinal lifting with a cystic appearance.

**Figure 3.**
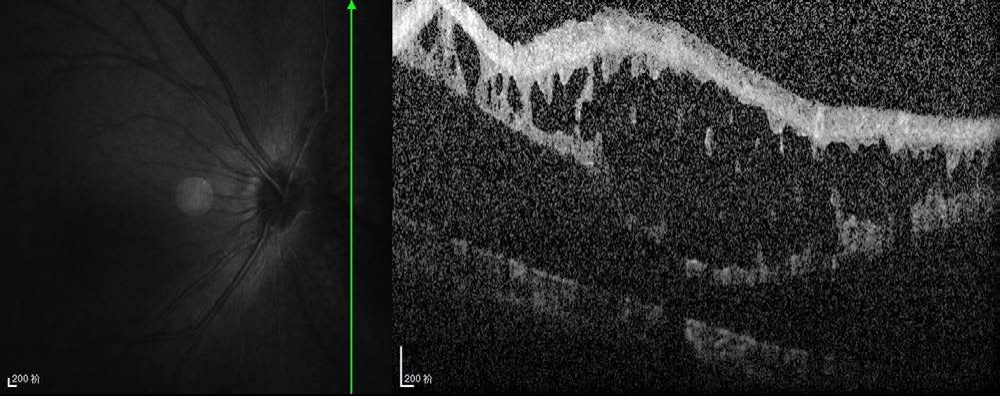
Optical coherence tomography of the optic nerve showed peripapillary retinal detachment in the right eye.

**Figure 4.**
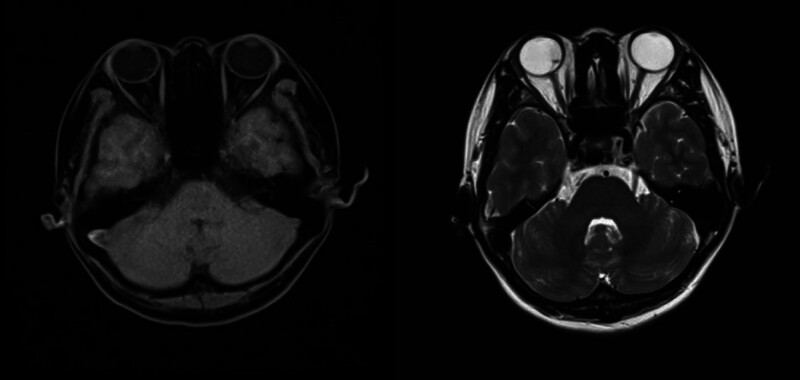
Orbital magnetic resonance imaging showed a long T1 and long T2 signal shadow on the nasal side of the lens level of the right eyeball, with a wide base attached to the wall of the optic globe.

**Figure 5.**
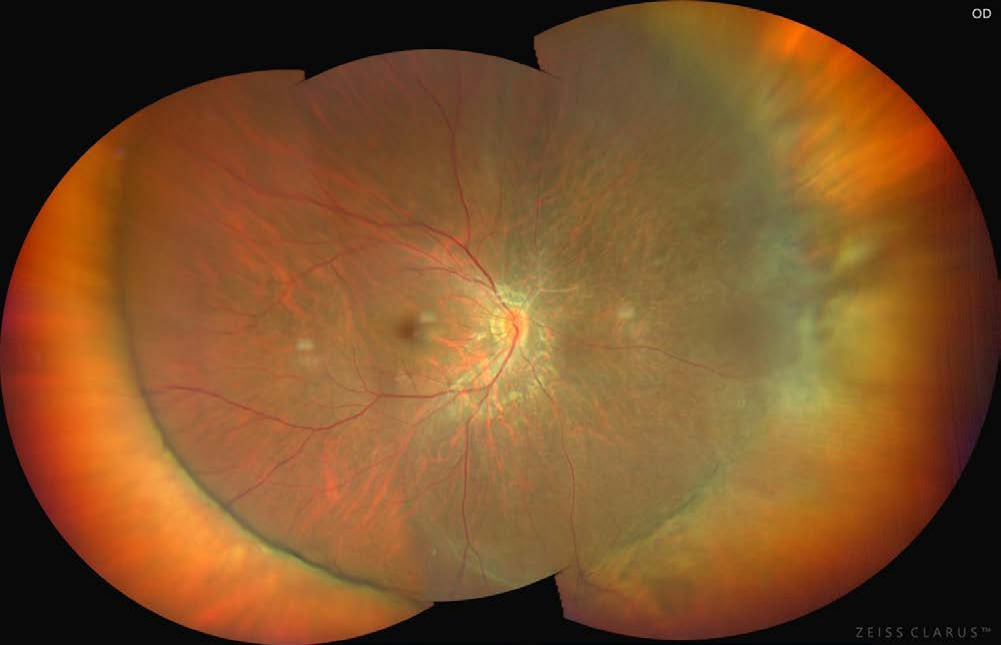
After scleral buckling with external compression combined with extracapsular drainage of intracapsular fluid, the patient’s retina was well restored.

A 32-year-old woman presented with dark shadows in the right eye for 3 consecutive days with decreased vision. She had no history of trauma or other illness. However, the patient had a brief history of severe coughing before the onset of vision loss. The best-corrected visual acuity in the right eye was 0.5. Slit-lamp biomicroscopy showed grade III anterior chamber blood cells and grade III vitreous hemorrhage in the right eye, clear borders of the optic disc, a reddish color, and an approximately normal retinal vessel diameter and alignment; a large cyst was visible on the nasal side of the retina, protruding into the vitreous cavity, with well-defined borders, peripheral retinal limitation of superficial detachment, and a large amount of hemorrhage vaguely visible within the cyst. The left eye was essentially normal. The intraocular pressure was 12 mm Hg in the right eye and 14 mm Hg in the left eye. The large cyst was located in the nasal region of the optic disc from 1:00 to 5:00 (Fig. 1). There was extensive well-defined retinal detachment at the posterior border of the cyst and massive hemorrhage within the cyst. B-scanning revealed vitreous clouding in both eyes, posterior vitreous detachment in the right eye, and retinal detachment in the right eye (cyst not excluded) (Fig. 2). Orbital magnetic resonance imaging showed a long T1 and long T2 signal shadow on the nasal side of the lens level of the right eyeball, with a wide base attached to the wall of the ball. The diagnostic imaging opinion was as follows: Consider detachment of the retina with subretinal effusion (Fig. 3). Ultrasound biomicroscopy showed a right anterior chamber axis depth of 2.60 mm, a left anterior chamber axis depth of 2.63 mm, and turbid aqueous humor in the right eye. Macular optical coherence tomography showed a diffuse reflex in the macular region of the right eye and nasal retinal detachment. Optical coherence tomography of the optic nerve showed peripapillary retinal detachment in the right eye (Fig. 4). The diagnosis was retinal detachment of the right eye, retinal cyst of the right eye, and retinal split of the right eye. After 3 months of blood circulation and other supportive treatments, the patient’s hemorrhage was significantly absorbed, but the size of the cyst did not change significantly. Consequently, the patient then underwent scleral ring ligation with external pressure combined with external drainage of the intracapsular fluid; however, we did not find any retinal clefts preoperatively or intraoperatively, the patient’s right best-corrected visual acuity was restored to 1.0 after the surgery, and the retina was well restored (Fig. 5).

## 3. Discussion and conclusions

The term retinal cyst was introduced in 1963 by Pischel.^[3]^ He classified such cysts into 4 types: small cysts: approximately 1 mm in size; intermediate or isolated cysts: 4 to 8 PD in size; giant cysts: 8 to 10 PD in size; and large retinoschisis: this type of cyst is larger than (3) and occupies at least 25% of the fundus. Pische’s analysis also suggested that retinal cysts larger than 4 PD in diameter or larger than 1 mm with flashing lights and blackout sensations were of therapeutic interest and recommended treatment including scleral electrocoagulation, laser photocoagulation, scleral puncture, or a combination of these methods. The cyst in this case was approximately 10 PD in size, so it was more appropriately called a giant cyst. On B-scan, the retinal cyst was seen as a typical cystic echogenic area located on the retinal detachment band, and the retinal detachment was associated with hemorrhage.^[4]^ In cases of retinal detachment with hemorrhagic cysts, which are less common, B-scanning reveals a strongly echogenic band of a detached retina, on which the cysts are more often rounded, and the internal echoes are different from those of ordinary cysts, which are echo-less, due to the presence of hematopoietic cells; moreover, the concentration of hemorrhage may be manifested in the form of strips of strong echoes.^[5]^ Many researchers have since highlighted the relationship between intraretinal cysts and long-term retinal detachment. It is generally accepted that retinal cysts are secondary to rhegmatogenous retinal detachment, and the duration of the disease is usually more than 3 months, but in the present patient, the duration of retinal detachment was shorter by only 3 days. Similarly, this patient did not have retinal tears and mainly showed retinal splitting with a shallow detachment that was confined around the cyst demarcation line. She also had a brief history of severe coughing before the onset of vision loss, and we hypothesized that her condition may have been due to the presence of a small retinal cyst prior to the severe coughing, which was stimulated by severe coughing and resulted in enlargement of the cyst and rupture of the cyst and hemorrhage. Alexander et al^[6]^ also reported a case of retinoschisis with intraretinal cyst hemorrhage due to roller coaster riding. Keith^[1]^ and Sen and Mishra^[7]^ reported a similar case of retinal cleavage with a large retinal cyst. In 1966, by studying the pathological sections of 144 eyes, Keith^[1]^ classified retinal cysts as congenital and acquired; he found that the etiology of acquired retinal cyst included choroiditis, vascular injury, ocular trauma, and retinal detachment, etc, and surmised that its pathology progressed as follows: cystic degeneration caused by damage such as hypoxia of the retina and formation of small cystic cavities, which further fused to form a retinoschisis and ultimately progressed to retinal cyst. There have also been reports of cases where retinal cysts can be secondary to systemic amebas, *Klebsiella, Echinococcus* infections, and cerebral cysticercosis.^[8–10]^ Currently, the treatment of retinal cysts has been reported to be inconsistent. Cai et al^[11]^ concluded that vitrectomy combined with retinotomy is very effective in removing large cysts and providing adequate relief from retinal traction, but Brent et al^[6]^ emphasized that there is still a place for conservative treatment. This case report describes the use of scleral buckling with external compression combined with external drainage of intracystic fluid for the treatment of a large intraretinal cyst with retinal cleavage. Initially, this patient showed no significant change in the cyst after 3 months of conservative treatment; then, after scleral buckling with external compression combined with extracapsular drainage of intracapsular fluid, the patient’s retina was well restored. Scleral buckling allows flattening of the retina with essentially no disturbance of the vitreous, while external scleral drainage under direct observation allows for more complete removal of subretinal and intracystic fluid and avoids the risk of medical rupture of the highly mobile retina. In addition, drainage of subretinal fluid containing retinal pigment epithelial cells and other cytokines from the outside could theoretically prevent this inflammation by preventing this environment from entering the vitreous cavity and inner surface of the retina to reduce the risk of proliferative vitreoretinopathy. This is why the external approach has the advantages of being less invasive, yielding fewer complications, and avoiding secondary surgery. We are well aware that external drainage of intraretinal cyst fluid without causing medical retinal tears is the key to successful surgery. It is worth noting that in the previous literature, vitrectomy was often used to remove cysts in such patients, with more surgical complications, whereas in our patient, an external approach was used; there have been no reports on the use of scleral buckling with external compression combined with external drainage of intracapsular fluid to treat retinal cysts, which provides a new way of considering and approaching the treatment of subsequent cases.

## Author contributions

**Conceptualization:** Yanhui Wang.

**Data curation:** Jing An.

**Funding acquisition:** Xiaolu Cao, Lifei Wang.

**Investigation:** Yifan Wang, Peipei Jia, Jianan Liu.

**Methodology:** Yifan Wang, Jianan Liu.

**Project administration:** Peipei Jia, Lifei Yuan, Jing An, Lifei Wang.

**Resources:** Xiaolu Cao.

**Supervision:** Lifei Yuan, Yanhui Wang, Lifei Wang.

**Writing – original draft:** Yifan Wang.

**Writing – review & editing:** Yifan Wang, Xiaolu Cao, Lifei Yuan, Yanhui Wang, Lifei Wang..
